# Utilization of a population health survey in policy and practice: a case study

**DOI:** 10.1186/1478-4505-11-4

**Published:** 2013-01-30

**Authors:** Rachel Laws, Lesley King, Louise L Hardy, Andrew Milat, Chris Rissel, Robyn Newson, Lucie Rychetnik, Adrian E Bauman

**Affiliations:** 1 Prevention Research Collaboration, School of Public Health, University of Sydney, Sydney, 2006, NSW, Australia; 2 School of Public Health, University of Sydney, Sydney, 2006, NSW, Australia

**Keywords:** Government, Policy, Population health, Research

## Abstract

**Background:**

There is growing interest by funding bodies and researchers in assessing the impact of research on real world policy and practice. Population health monitoring surveys provide an important source of data on the prevalence and patterns of health problems, but few empirical studies have explored if and how such data is used to influence policy or practice decisions. Here we provide a case study analysis of how the findings from an Australian population monitoring survey series of children’s weight and weight-related behaviors (Schools Physical Activity and Nutrition Survey (SPANS)) have been used, and the key facilitators and barriers to their utilization.

**Methods:**

Data collection included semi-structured interviews with the chief investigators (n = 3) and end-users (n = 9) of SPANS data to explore if, how and under what circumstances the survey findings had been used, bibliometric analysis and verification using documentary evidence. Data analysis involved thematic coding of interview data and triangulation with other data sources to produce case summaries of policy and practice impacts for each of the three survey years (1997, 2004, 2010). Case summaries were then reviewed and discussed by the authors to distil key themes on if, how and why the SPANS findings had been used to guide policy and practice.

**Results:**

We found that the survey findings were used for agenda setting (raising awareness of issues), identifying areas and target groups for interventions, informing new policies, and supporting and justifying existing policies and programs across a range of sectors. Reported factors influencing use of the findings were: i) the perceived credibility of survey findings; ii) dissemination strategies used; and, iii) a range of contextual factors.

**Conclusions:**

Using a novel approach, our case study provides important new insights into how and under what circumstances population health monitoring data can be used to influence real world policy and practice. The findings highlight the importance of population monitoring programs being conducted by independent credible agencies, researchers engaging end-users from the inception of survey programs and utilizing existing policy networks and structures, and using a range of strategies to disseminate the findings that go beyond traditional peer review publications.

## Background

Public funds are expended through health research to lead to improvements in policy [1,2], practice, resource allocation and, ultimately, the health of the community [3]. This can only occur if the evidence derived from the research is used to inform practice and policy decisions. There is growing interest by both funding bodies and researchers in measuring the impact of research. Over the past two decades many theoretical frameworks and approaches to measuring research impacts have been proposed [4], but there have been few empirical studies exploring how and why research is used [5,6].

Population health monitoring surveys form one component in the public health research cycle, providing a key source of information about the prevalence and patterns of public health problems [7]. Such information may assist in guiding appropriate interventions, track changes over time, and support evaluation processes. That is, population monitoring surveys potentially provide valuable information to support policy and practice, although further research is generally required to test and disseminate effective interventions [8].

Despite the high investment in monitoring, little is known about if and how this type of population health data is used to inform policy and practice or the key factors influencing its use. We identified only one such study by de Goede and colleagues, exploring the use of epidemiological research in the development of local public health policy in the Netherlands [9,10]. This study found that survey data was more often used in a conceptual way to improve understanding of the health problem or issue, rather than in a specific and direct way (instrumental use) or to justify a position or particular course of action (symbolic use) [10]. Research use was influenced by interaction between researchers and local health officials, the personal belief systems of the actors involved, and a range of contextual factors [9].

Further research is required to explore if, and in what ways, population health monitoring data is used in other contexts and settings. Understanding the factors influencing the use of this type of research is important in informing how population health monitoring systems can be planned and implemented to enhance the uptake of findings into the policy and program planning process.

Therefore, the aim of this paper is to provide a case study analysis of the utilization of the findings from an Australian series of population monitoring surveys of school-aged children’s weight and weight-related behaviors, the Schools’ Physical Activity and Nutrition Survey (SPANS). Three surveys have been conducted in 1997, 2004 and 2010 in the state of New South Wales (NSW), and provide a key source of data on NSW school children’s weight status, physical activity, sedentary behaviors and nutrition [11-13]. Funded by government educational and health agencies since 1997, this monitoring program provides information on changes over time in key variables, and can potentially contribute to guiding and monitoring the progress of public health policy and programs that address children’s weight-related behaviors. However, the extent to which the surveys have actually been used for these and related purposes, has not yet been systematically investigated or documented.

The specific aims of this paper are to: i) describe if and how SPANS findings have been used at various time points to influence policy and practice; ii) explore the key facilitators and barriers to the use of the findings; and, iii) critically reflect on the methods applied to assess research use and make recommendations for future studies.

## Methods

**Figure 1:**
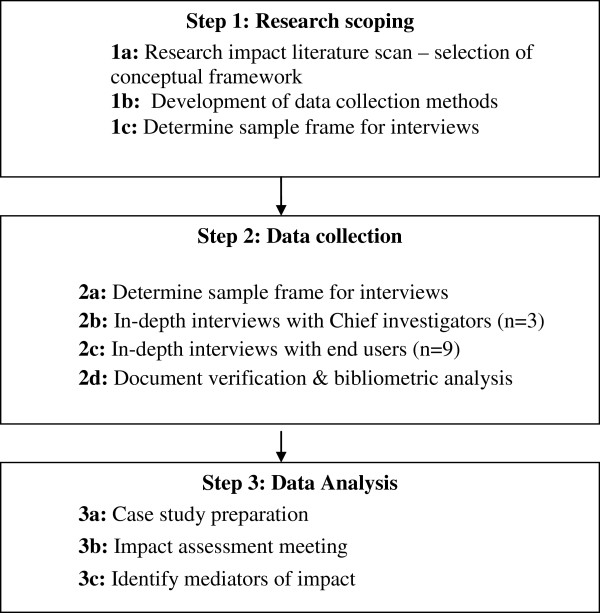
Overview study methods and key steps in the research process.

This study utilized a case study approach to explore if, and in what ways, a population health monitoring program was used to influence policy and practice, and to identify the key factors influencing its use. Case study methods are appropriate for answering ‘how’ and ‘why’ questions when the phenomenon of interest (SPANS surveys) is embedded within a real-life context (policy and practice environment) [14]. The study was approved by the University of Sydney Human Research Ethics Committee and all participants gave written informed consent to take part in the study. An overview of the study methods and key steps in the research process are given in Figure 1.

### Step 1 - Research scoping

The impact of SPANS was examined using the theoretical framework proposed by Banzi *et al.* based on a systematic review of available models [4]. The framework has five broad impact categories: i) Advancing knowledge and research related impacts (peer review articles, impact on research methods, better targeting for future research); ii) Capacity building (development of research capacity of staff, students, others); iii) Informing policies and product development (policy, guidelines, product, intervention development); iv) Health and health sector benefits (improvements in service delivery, effectiveness of services, equity of services, cost reductions, etc.); and, v) Societal and economic impacts (e.g., improvements in health status, social benefits, shift in knowledge, attitudes, behaviors, social capital, macroeconomic).

The Banzi framework was chosen as it is based on a systematic review of existing models and the set of categories provide an integrated ‘map’ of a broad range of potential areas of impact, which largely reflect the range of other commonly used models, for example, the payback framework [15]. We will briefly report on the impact of SPANS in relation to categories one and two (advancing knowledge and capacity building), and provide more detailed results relating to practice, policy and broader impacts (categories three to five), as well as reporting on the key mediators of these impacts.

### Step 2 - Data collection

#### Chief investigator and end-user interviews

Semi-structured interviews were conducted with the Chief Investigators (CIs) for each of the three surveys (1997, 2004, 2010) and relevant end-users (EUs) including policymakers and practitioners who were in positions to make decisions regarding programs and policies related to SPANS findings on school-aged children’s nutrition, physical activity, sedentary behavior and obesity prevention in NSW. Both the CIs and EUs were invited to participate in the interviews by email, with non-responders sent a reminder email after one week and then followed-up by telephone. Interviews were conducted by an experienced research officer (RN) who had a good working knowledge of SPANS and related policy context, but was independent of the SPANS investigators. All interviews were tape recorded with participants’ permission.

The CI interviews explored their perspectives on the overall impacts of the survey, asked about specific impacts in accordance with the categories outlined in the Banzi framework, and any factors contributing to such impacts or lack thereof (List 1). The CIs were also asked to nominate up to three end-users (EUs), defined as ‘individuals who could provide a perspective on how the monitoring data had been used in policy, practice, organizational development, further research or in applications such as guidelines or teaching materials’. Attempts were made to identify EUs from a range of sectors including health, education and sport and recreation. These EUs were then approached and invited to participate in an interview exploring how the SPANS program findings had been used from their perspective.

List 1. Semi-structured interview topic guide: investigators and end-users

· Recall of research aims, key findings and implications

· Dissemination process (how, factors influencing the dissemination process)

· Interface with end-users – how research team worked with potential end-users (investigators only)

· Interface with researchers – how were end-users involved in the research project, how did they hear about the findings (end-users only)

· Overall impact – how have the findings been used

· Specific impacts – capacity building, partnerships, policy and product development, health and other sector impacts, societal and economic impacts

· Circumstances surrounding the use of the findings, or limited impact of the findings

· Evidence of impacts – documentary sources

· Nomination of end-users (investigators only)

#### Bibliometric and document analysis

A bibliometric analysis was undertaken in Scopus database to examine the total and mean number of citations (excluding self citations) for all peer review publications arising from SPANS for each survey year. Survey reports available in the public domain were also examined to document the key findings and recommendations arising from SPANS. CIs and EUs were also asked to provide a copy of any documentary sources which provided evidence of how SPANS findings had been used, such as policy documents, briefs, reports and curriculum materials. Additional searches of the grey literature were undertaken to identify documentary evidence of impacts identified in the interviews.

### Step 3 - Data analysis

All interviews were transcribed verbatim and coded by one author (RL) using Nvivo qualitative software program (NVivo (version 9) Burlington, MA, USA). The Banzi categories were adapted and expanded for the coding framework to encompass other categories of impact arising during the interviews and to code for important contextual information. Further, additional codes were developed to capture content related to key factors influencing research use.

Detailed case reports for each survey (1997, 2004 and 2010) were compiled. These consisted of a summary of: i) key SPANS survey findings and recommendations; ii) the perspectives of CIs and EU on how the survey findings had been used and key factors influencing their use; iii) bibliometric analysis; iv) documentary evidence of impacts; and, v) notes and observations made during CI and EU interviews. The case summaries were prepared by two authors (RL and RN), who were not involved in either the implementation or administration of the SPANS surveys, in order to ensure that the ‘insider’ experiences of three of the authors who were involved in one or more surveys (LLH,LK, AB) were not unduly influential.

The three case summaries were then independently reviewed by each of the authors. There was high consistency in findings across surveys, allowing for the distillation of key themes across all three surveys, covering how and why SPANS findings had been used in policy and practice. Attention was also paid to examining any differences between surveys, the perspectives of CIs and EU, and key contextual factors.

## Results

### Participant characteristics

All three of the survey CIs approached agreed to participate in the interview. A total of 14 EUs were nominated and approached, and nine agreed to participate. Of the non-participant EUs, two did not respond to the invitation, two were not available during the data collection period and one had moved on from the policy area and therefore did not feel able to comment on the survey impacts. The EUs worked in a variety of sectors including education, health, and sport and recreation, and in most cases had direct responsibility for issues related to child obesity prevention (Table 1). A total of 16 interviews were conducted, with CI interviews lasting longer (mean; 64 minutes; range 53 to 70 minutes) than EU interviews (mean; 50 minutes; range 17 to 87 minutes). Not all CIs and EUs were in a position to comment on the impact of each survey. Table 2 shows CI and EU participants by sector, role and survey year.

**Table 1:** Participant characteristics by sector and survey

** Participant**	** Participant sector and role**	**Survey**		
		**1997**	**2004**	**2010**
CI 1	Academic researcher	✓	✓	
CI 2	Academic researcher		✓	
CI 3	Academic researcher			✓
EU 1	Health^1,2,3,4^	✓	✓	
EU 2	Community sport and recreation^2,3,4^		✓	✓
EU 3	Education^3,4^		✓	✓
EU 4	Education^4^			✓
EU 5	Education^4^			✓
EU 6	Health^1,2,3,4^		✓	
EU 7	Health^4^		✓	
EU 8	Health^4^			✓
EU 9	Health^1,2,3,4^			✓
**Total number of interviews**	**12**	**2**	**7**	**7**
	**CI interviews**: **3**	(**1 CI**	(**2 CIs**	(**1 CI**
	**EU interviews**: **9**	**1 EU**)	**5 EUs**)	**6 EUs**)

^1^Worked in policy branch responsible for funding SPANS.

^2^Member of at least 1 SPANS Advisory Committee.

^3^Member of Government Child Obesity Prevention Implementation Committee.

^4^SPANS information relevant to work area.

**Table 2:** **Key findings**, **implications and dissemination process for the SPANS monitoring program**

**Key Findings**	**Key Findings**	**Dissemination Process**	**Funding Source**
**1997 School Fitness and Physical Activity Survey**			
Poor fundamental movement skills (FMS) amongst school students	Need to address FMS and physical activity in schools	Main and summary report available on website	Department of Education and Training
Low levels of physical activity, particularly amongst adolescent girls	Survey should be repeated periodically with comparable measures	Summary report sent to all NSW schools	
Few primary schools have teachers with training in sport and physical education		Presentations to a range of end-user groups	
		Peer review publications (n = 11, total citations^1^ = 589)	
**2004 School Physical Activity and Nutrition Survey**			
Almost 25% of students 5–16 years were overweight or obese, rising from 1997	Focus on FMS working, but needs to be continued	Main, short and summary report available on website	NSW Department of Health
Widening of the gap in prevalence of overweight and obesity amongst low socio-economic status and culturally and linguistically diverse groups since 1997	Need to limit small screen use to 2 hours per day	40 Regional stakeholder workshops across NSW	
FMS and physical activity levels had improved significantly since 1997 survey but high screen time use	Reinforced the need to implement government policy that schools should offer 2 hours of planned physical activity per week	Media releases and interviews	
Chronic disease risk factors common amongst overweigh/obese adolescents	Efforts to increase physical activity promotion in schools and community should be continued and increased	Peer review publications (n = 18, total citations^1^ = 131)	
High intake of energy dense foods and drinks and low levels of vegetable consumption	Limit consumption and promotion of energy dense nutrient poor food		
	Implement strategies to target ‘high-risk’ groups		
**2010 School Physical Activity and Nutrition Survey**			
Prevalence of overweight and obesity stabilized since 2004	Continue to implement school canteen program, focus on FMS and provide minimum of 2 hours of planned physical activity per week in schools	Main, short and summary report available on website	NSW Department of Health (now Ministry of Health)
Socioeconomic and cultural disparities in prevalence of overweight/obesity remain.	Implement programs to support participation in community sport	Media releases and interviews	
High consumption of energy dense nutrient poor foods and drinks and low levels of vegetable consumption	Advocate for national regulations to limit the marketing of unhealthy foods to children and policies to promote active transport	Peer review publications (n = 4, total citations^1^ = 1)	
Decline in proportion of students meeting physical activity guidelines	Widespread dissemination of consistent messages to parents/families regarding healthy lifestyle behaviors	Presentations to stakeholder groups (with more to follow)	
FMS showed improvement in some skills and decline in others	Importance of targeting family behaviors and early childhood sector to reduce obesity in preschoolers		
High screen time use			
Overweight and obesity increasing among children entering first year of school (~ 5 years old)			

^1^Excluding self citations.

### Research impacts

#### Dissemination and advancing knowledge

In general, the survey findings were considered to advance knowledge through the findings being widely reported in journal articles, and extensively disseminated through key reports, presentations to stakeholder groups, media releases and conference presentations. In total there were 32 peer review publications (1997, n = 11; 2004, n = 18; and 2010 n = 3, at the time of this analysis - July 2012) which together had been cited 720 times (Scopus, excluding self citations) with a mean of 22 citations per article (SD: 34, range: 0 to 140, H index: 12: Scopus). The key findings and recommendations from each survey, along with the dissemination process that was employed and survey funding source are presented in Table 2. While dissemination processes varied for each survey, in each case they included presentations to health and education professionals (mostly health promotion staff and teachers), as well as senior NSW policymakers in health, education, sports and community sectors.

Almost all EUs reported that they had direct involvement in the administration or funding of the survey from its inception as well as the dissemination process, including the drafting of key recommendations. Some EUs also took an active role in disseminating the findings within their sector, as discussed by this EU:

*“…after each of the SPANS surveys that came out… we had these workshops… so we were provided the data and the information to our industry, but tried to do it through a meaningful way so they can apply it when they go into schools themselves.”* (EU2, 2004 and 2010 surveys).

CIs and EUs discussed that the dissemination of SPANS findings was important in raising overall awareness of the issue of child obesity amongst the general community and key stakeholders such the education, health, and sport and recreation sectors, as well as highlighting areas for intervention and future research.

#### Capacity building impacts

The surveys had considerable impact on building research capacity with two PhDs, two masters/honors research projects and two postdoctoral positions arising from SPANS. For EU’s the survey provided professional development opportunities for the 55 school teachers who were involved in data collection, including up-skilling in the measurement of fundamental movement skills, cardiorespiratory fitness and anthropometry. Further, the survey methodology was used to inform the methods for other research studies and survey programs at both the state and national level (Additional file 1).

#### Policy and practice impacts

Table 3 presents the key content themes regarding ways in which the survey findings were used to inform policy and practice, as well as illustrative quotes. At a policy level, this ranged from broad agenda setting and policy debates on the importance of childhood obesity, to underpinning specific new policies such as banning soft drink sales in schools. Each survey had specific policy impacts (e.g., 1997 findings informed the development of a policy to focus on fundamental movement skills (FMS) in schools, and 2004 findings underpinned the ban of soft drink sales in schools). The EUs and CIs identified a range of policy documents which cited or applied SPANS findings (Additional file 1).

**Table 3:** How the SPANS program findings were used to inform policy and practice

**Key Impacts**	**Illustrative Quotes**
***Policy Impacts***	
· **Agenda and priority setting** e.g., attracting funding to the issue of child obesity prevention, identifying priority groups and settings for intervention	“*I guess it sort of drives*, *to some degree*, *the priorities in the* [*government obesity*] *plan*.. *are the actions in the plan the right ones*, *are they working*?…*So it gives us a bit of* …*an idea of whether or not there*’*s gaps that haven*’*t been addressed*, *in terms of the broader cross government strategy*.. *are there areas where we could pull back on*, *invest more*, *focus in another area.”* (EU9, 2010 survey)
· **Informed policy debates** e.g., data used in briefings with health minister to inform parliamentary debates	
· **Informed policy planning** e.g., identifying areas for investment, de-investment and stakeholder involvement in government obesity plan	“….*there*’*s high levels of screen time*, *so I guess we can kind of use it as a basis or rationale for our policy input*, *and also for how we approach different issues with different government agencies.”* (EU9, 2010 survey)
· **Directly underpinned new policy** e.g., banning soft drink sales in schools	
	*“I know that the physical activity components* [*1997 survey*] *were also used to advocate for fundamental movement skill development with the Department of Education.”* (EU1, 1997 & 2004 surveys)
· **Indirectly used to advocate for new policy** e.g., food marketing to children	
	*“SPANS would certainly have been one of the things that contributed to the development of that policy* [*nutrition in schools*].*”* (EU5, 2010 survey)
· **Used to support existing policy** e.g., after school physical activity programs for children	
	“*the stats that are so terribly important*, *particularly when you*’*re trying to sell something*” (EU4, 2010 survey)
· **Policy evaluation** e.g., performance monitoring tool for government obesity plan	*“And the Education Department was particularly interested to see what had happened in terms of fundamental movement skills*, *because they*’*d made a significant investment in fundamental movement skill education in primary and high schools*, *so they were really interested in what had happened there*. *And another part of it was an evaluation of the Healthy Canteen strategy that had been put in place*, *maybe a year or two earlier*. *And there was also an aspect of the study that was looking at the school environment to see again if there had been any changes following the previous study*.*”* (CI3, 2004 survey)
	“*SPANS just shines a light on what*’*s not working.”* (EU2, 2004 & 2010 surveys)
***Practice Impacts***	
*School Sector*	“…*the initial research that was done* [*1997 survey*]…*we got very strong agreement around those fundamental movement skills*, *and we had some clear support materials developed for schools and the Get Skilled Get Active DVD*, *which is a resource that we still promote in schools.*” (EU3, 2004 & 2010 surveys)
· **Informed curriculum development** e.g., standardized approach to teaching FMS	
· **Lead to new curriculum resources** e.g., standardized teaching materials for FMS	
· **Informed Professional development for teachers** e.g., measuring FMS, raising awareness of child obesity	
· **Informed and supported existing health promotion programs** e.g., healthy canteens	
*Sport and Recreation Sector*	“…*the* [*sport*] *development officer network*, *they loved it* [*survey data*] *and they got it*. *Particularly the fundamental movement skills*, *because they*’*re out there teaching them*, *through sport*, *every day*… *they*’*ve now rescheduled their format of how they go in to teach girls Rugby* …*they do the kicking last because they know their stronger skill is in the running and the catching*. *So*, *it*’*s really practical*…” (EU2, 2004 & 2010 surveys)
· **Informed sports coaching programs** e.g., how FMS are taught in some sports	
· **Supported rationale for new programs or pilots** e.g., healthy sports canteens	
*Cross Government* / *Community Sector*	“…*when it* [*2004 survey*] *showed that 20*% *of kids when they started grade 1 were already fat*, *it also gave emphasis to do stuff in childcare*…*that was really compelling*…*we would have never known that if that survey wasn*’*t done.*” (EU6, 2004 survey)
· **Informed development and refinement of educational resources** e.g., website on healthy living for parents and general community [35]	
	“*SPANS*, *as I said*, *is a moment in time that gives you the heads*-*up on what areas you should be focusing on in you next plan*; *that*’*s what it should be doing.*” (EU2, 2004 & 2010 surveys)
*Health Sector*	“*I think SPANS contributed to having treatment programs is what they are* – *I call them treatment programs for people with already* – *with* – *with already with the problem.*” (EU6, 2004)
· **Informed program planning** e.g., choice of target groups and settings for intervention and availability of treatment programs	

At the practice level, SPANS findings were used to inform program planning across a range of sectors. In the education sector, the findings led to a standardized approach to teaching FMS in schools and underpinned the rationale for new initiatives in the community sports sector (such as healthy sports canteens). The findings provided support for some existing health promotion programs, and informed the development and refinement of educational resources both within schools and for the broader community. The impact on practices across sectors largely flowed on from policy impacts. For example, the policy focus on FMS in schools led to the development of new curriculum materials, resources and professional development for teachers (Additional file 1).

#### Broader health, economic and societal impacts

Most interview respondents found it difficult to comment on the broader health, economic or societal impacts of SPANS, because of the long term nature and the multiple factors contributing to such impacts. Nevertheless, one EU suggested that SPANS may have contributed to improvement in FMS over time because of the change in curriculum focus and resources provided to schools following the 1997survey. Others discussed that SPANS may have contributed indirectly to the leveling-off of childhood obesity between 2004 and 2010, as new policies and programs arose, in part, because of the survey findings.

*“I think the fact that obesity has plateaued should be taken that all of the investments that have been happening…well at least since SPANS 2004…are obviously having some degree of impact”* (CI 3).

Documentary checks were undertaken and documentary evidence identified relating to the majority of documents and initiatives referred to by CIs and EUs. Key documents are listed in the Additional file 1.

### Factors influencing the use of SPANS findings to inform policy and practice

An analysis of key perceived facilitators and impediments to the use of the SPANS findings in influencing policy and practice revealed three main inter-related factors: i) the perceived credibility and trustworthiness of the survey findings; ii) the dissemination processes used; and, iii) a range of contextual factors (illustrative quotes about these factors are presented in Table 4). In 1997, the survey was funded by government as part of a broad child health and fitness agenda, and from 2004, as part of a government policy response to monitor child obesity. As such the survey content and findings were designed to be closely aligned to the interest of policy makers and key EU groups such as health, education, and community sport and recreation sectors. The survey was perceived by EUs interviewed to be a trustworthy source of data as it was conducted by an independent and credible research group.

**Table 4:** Factors influencing the use of the SPANS findings to inform policy and practice

**Facilitators**	**Barriers**
***Research Quality and Content***	
· **Survey program perceived to be of high methodological quality**: longitudinal and conducted by an independent and credible research team	· **Survey findings did not provide all the answers:** needed to be considered alongside evidence about effective interventions
	“…*of course just monitoring is not a solution*” (EU6, 2004)
· **Survey program perceived to be aligned to the priorities of policy makers and practitioners** with adaptations made over time to meet needs	**Lack of specificity of the data:** Unable to provide data at regional/sector level
	“*I think SPANS cannot stand alone*, *it has to have an evidence summary of interventions behind it to inform good policy*” (EU2, SPANS 2004 & 2010)
“*the Ministry*/*Department has worked really hard*…*to make sure that*…*the questions that the survey*’*s asking are*, *I suppose*, *the right ones for the priority areas as well*…*So I think over time the surveys have kind of adapted to have different kinds of questions in them*…*it*’*s been purposefully closely aligned with policy priorities and government priorities and I think that helps*” (EU9, 2010 survey)	
***Dissemination Process***	
· **Use of active dissemination strategies** e.g., discussion of findings at workshops between researchers and end-users.	**Lack of reports tailored to specific end-user groups**/**sectors** highlighting key implications of the findings
“… *there was a major dissemination strategy amongst a set of orgs*” (EU6, 2004)	
	“…*there probably should be a report for practitioners* … *What does this mean to you*?”…
· **A range of** ‘**knowledge transfer**’ **products produced** e.g., short reports highlighting key findings and recommendations.	*What does it mean for a teacher who*’*s 55*, *has never played sport before*..*I think if we really want to get good at this*, *take SPANS and write it for different audiences*… *So SPANS for academia*, *SPANS for policy*, *SPANS for the general public*” (EU2, 2004 & 2010 surveys)
· **End-users acted as** ‘**knowledge brokers**’ facilitating dissemination of SPANS findings within their sector	
“….*we*’*ve done canteen newsletters and things like that and we*’*ve reported SPANS results and we reference it there*” (EU5, 2010)	
· **Active engagement of the media** resulting in high levels of media coverage, public debate and discussion	
“*I also sit on the* [*policy*] *group for obesity*, *so the report was discussed on a number of occasions at that meeting as well*. *You see the media reports they*’*ve generated*…*we receive*…*draft copies of the report as well*, *in terms of the discussions that we had with Health*” (EU5, 2010 survey)	
***Contextual Issues***	
· **Supportive policy context** for addressing child obesity with the release of the SPANS findings fitting well with some policy cycles (government obesity plan and planning of national curriculum)	***Political instability and poor timing*** e.g., frequent changes in ministerial positions and poor fit with some policy cycles
	***Limited sector capacity and resources*** e.g., lack of funds to implement the findings in some sectors at certain time points
· ***Continuity and Partnerships between researchers and end-users*** from the inception of SPANS program facilitated dissemination, ownership and use of the findings	
	“….*then there*’*s some infrastructure issues we need to look at*; *there needs to be some resourcing*” (EU2, 2004 & 2010 surveys)
· ***Mechanisms and structures in place to implement recommendations*** e.g., Policy relevant forums involving key end-users	“…*probably prior to 2002* …*there was a relatively limited amount of funding in this aspect of Health Promotion* - …*So in terms of influencing programs it would have been difficult* ‘*cause we*’*ve* …*no money to include programs*” (EU1, 1997 & 2004 surveys)
· ***Good fit with organizational culture and ways of working*** e.g., value placed on having an ‘evidence base’ for practice and policy decisions amongst end-user groups	
“*I think the Department of Education knew of the findings or the recommendations that were going to be made because they also had ownership of those*..*they had to agree on those for the draft report*. *So I think by that stage they*’*d already said*: *We want this recommendation here and this is how we*’*re going to respond to it*.” (CI, 1997 & 2004 surveys)	

Partnerships between EU groups and the CIs operated from the inception of the SPANS program, and more regular and specific policy forums were established as part of the NSW ‘whole of government’ approach to addressing child obesity in 2003 [16]. These policy forums provided the continuity and mechanisms for survey findings to be disseminated and considered, and for EU groups to become engaged in formulating the key policy recommendations and the wider dissemination processes. There were, however, some barriers identified to the use of the findings, including some related to the nature of the survey itself, in that it mainly identified the problems and target groups, but did not provide specific guidance on solutions, and the reports were not written for specific EU groups. A number of contextual factors were also identified as impediments to applying the findings, including ongoing organizational changes in government agencies, and limited capacity and resources to implement recommendations among some sectors at various points in time.

## Discussion

This is one of the few studies to document the temporal impact of a government population health monitoring program. We found that SPANS was successful in advancing knowledge and understanding of the issue of child obesity, weight-related behaviors, building capacity of the academic researchers and some EUs, and influencing policy and practice decisions across a range of sectors. The actual policy and practice impacts were in line with key recommendations arising from the survey reports. The positive impacts of the survey findings were attributed to the perceived credibility of the survey by EU groups, the processes used to disseminate the findings and a range of contextual factors. Contextually, the survey had a supportive government policy, strong partnerships between CIs and EUs, and established mechanisms for dissemination of the findings across a range of government sectors.

This case study demonstrates the positive impact a population health monitoring program can have on a range of policy and practice decisions. In particular, the findings of this study have demonstrated that population health monitoring data can be used as an advocacy tool (to attract attention and funding to an issue), priority setting (identifying areas and target groups for intervention), support and justify existing programs/approaches, or identify the need for alternatives. While others have suggested that epidemiological research can be used in a conceptual way to improve understanding of a health problem [9,10], this case study found that the survey findings were also used instrumentally to directly underpin new policies (such as banning soft drink sales in schools), and symbolically to justify existing policies and programs (such as introducing healthy school canteens), in addition to improving understanding of the issues associated with child obesity (conceptual use).

The instrumental and symbolic use of the survey findings in influencing policy decisions may reflect the fact that the EUs interviewed in this study were directly involved in the development and implementation of policies and programs relevant to the survey findings. In contrast, the study by de Goede and colleagues involved a broader range of EUs, most of whom were not directly involved in execution of health surveys. The use of the survey findings may also reflect the political imperative to take action to reduce child obesity, particularly in the early stage of child obesity prevention responses when there was an absence of other evidence on effective intervention approaches [17,18]. It is important to note, however, that epidemiological evidence in itself does not provide solutions to health problems, and needs to be considered alongside the broader body of evidence about effective intervention and policy approaches, as highlighted by many of the EUs in this study.

It is clear from this study that population monitoring surveys are one of many factors influencing public health policy and practice. In line with previous research [5,6,19-24], we found that a range of contextual factors were critical in facilitating the use of the survey findings. In particular, the 2002 NSW Child Obesity Summit [18] provided a supportive policy context which facilitated the formation of partnerships between survey researchers and EUs, and provided mechanisms and structures (such as the NSW Government Action Plan for Child Obesity Prevention 2003–2007, [16] and a Senior Officers Group with responsibility for overseeing implementation of this plan) which facilitated application of survey findings. In addition, ongoing media coverage of child obesity throughout this period reinforced the political and social relevance of these surveys [25].

The findings highlight the importance of researchers tapping into existing policy networks, structures and processes, utilizing policy windows and building partnerships and trust with policy makers and other EUs over time. This is in accordance with the findings of Innvær and colleagues who found that personal contact and timely relevance were the most commonly reported facilitators of research use in a systematic review of 24 studies of the use of evidence by health policy makers [22]. Additionally, our findings point to the importance of the dissemination process in influencing the use of findings from population health monitoring surveys, in particular, the use of active dissemination strategies, such as workshops and presentations, to encourage contact and interaction between academic researchers and EUs. The use of a range of other dissemination mechanisms such as short summary reports in lay language and media releases, as well as traditional academic publications, were all shown to be important in facilitating use of the findings by EUs. There is increasing emphasis from funding agencies on making research evidence readily available, however recent studies of public health research suggest that most dissemination activity beyond publishing academic papers appears to be undertaken in an *ad hoc*, unfunded fashion and that access to dissemination advice and support for researchers from funding agencies and academic institutions is lacking [26,27]. This suggests that more emphasis should be placed on funding and supporting a range of dissemination activities on behalf of both funding bodies and academic institutions themselves.

Traditionally, tools for measuring research impacts and assessing ‘research excellence’ are based on the number and significance of academic publications (such as bibliometrics and journal impact factors). However, there is growing interest in assessing actual impact on real world policy and practice. While a number of theoretical frameworks for assessing impact have been proposed [5,28-32], the findings of our study suggest that the impact categories proposed in such frameworks, including that of Banzi *et al.*[4], can capture the practical ways in which survey data is utilized. However, the findings also indicate that it can be difficult to attribute impacts to a single piece of research, particularly the longer term societal, health and economic impacts, which almost always arise for a complex array of contributing and contextual factors. This highlights the need for alternative ways of conceptualizing research ‘impacts’. The use of ‘contribution mapping’ as proposed by Kok and colleagues [33] may provide an alternative way forward. This approach aims to assess ‘contributions’ of research as opposed to ‘impacts’ and focuses on understanding how research and knowledge utilization processes evolve amongst the key actors involved. Further clarity is also required by researchers and funding agencies about the intended contribution that population health monitoring surveys are expected to make to policy and practice, in order to assess the degree to which this was achieved.

This study has a number of strengths and limitations. In terms of strengths, we assessed impacts using multiple methods, including bibliometric analysis, interviews with survey researchers and EUs, and documentary checks. This allowed for triangulation of data in the form of case summaries. The documentary checks lend confidence that the perspectives of the EUs are credible. While the SPANS reports have been disseminated widely across the NSW education sector, the full range of users, and their responses and application of findings, are unknown, although likely to be highly variable. It must be acknowledged that the EU interviewees were purposefully sampled on the basis of their role as the primary target audience and having direct responsibility for child obesity prevention policy initiatives. There may also have been some degree of social desirability response bias, whereby EUs felt obliged to report positive or over-inflated impacts. We attempted to reduce social desirability response bias by having researchers not previously involved in SPANS conducting the interviews and undertaking the analysis. The recall of impacts was somewhat problematic for the 1997 survey, as this was conducted over 15 years ago and only one EU (out of three) was available to be interviewed. Hence we are unable to ascertain whether the impacts of the 1997 survey may be underestimated or overestimated in this case study. Overall, there has been a high degree of continuity in the CIs involved in SPANS and EUs in NSW child obesity prevention over the study period, which may also have increased difficulties in attributing influence to any single one of the three surveys, and lent a positive bias. We recognize that there is no single or straightforward way of determining the full range of impacts across multiple potential EUs; however this case study provides a practical illustration of how a Government population survey can be used.

## Conclusions

The SPANS case study provides important new insights into how population health monitoring data can be used to influence policy and practice. This ranged from raising awareness of the issue (i.e., child obesity), identifying priority areas and target groups for interventions, underpinning new policies and supporting and justifying existing policy and/or programs. Our findings highlight the importance of survey monitoring programs being conducted by credible independent agencies, the use of a range of dissemination strategies that directly engages EUs, and the need for strong partnerships with policy makers and EUs from the inception in order to increase ownership over the findings and commitment to action. Population monitoring data, however, needs to be considered alongside other evidence about effective intervention approaches to maximize its influence on practice and policy decisions.

## Abbreviations

CIs: Chief investigators; EUs: End-users; SPANS: Schools’ Physical Activity and Nutrition Survey.

## Competing interests

Four of the authors of the current study (LLH, LK, AB, AM) were either CIs or contract managers of the SPANS surveys. In order to manage this potential conflict of interest, these individuals were excluded from conducting interviews with CIs or EUs and were also excluded from the production of case summaries which provided assessment of research impacts. The potential conflicts of interest were acknowledged by the individuals at the Impact Assessment Meeting and were factored into committee deliberations when designing this study.

## Authors’ contributions

RL, LK, LLH, CR and AM conceived the study and designed the methods. RN was responsible for conducting the interviews and collecting documentary sources. RL undertook analysis of the case study data, produced the case summaries and drafted the manuscript. All authors contributed to data interpretation and have read and approved the final manuscript.

## Supplementary Material

Additional file 1 **Documentary Evidence: List of reports and documents cited by CIs and EUs (excluded peer review publications)**. Click here for file
