# Supplementary material for: Utilization of a population health survey in policy and practice: a case study
Source: Health Res Policy Syst. 2013 Jan 30;11:4. doi: 10.1186/1478-4505-11-4 (PMC3564893; doi:10.1186/1478-4505-11-4)
Supplement: Additional file 1 — Documentary Evidence: List of reports and documents cited by CIs and EUs (excluded peer review publications). [file 1478-4505-11-4-S1.pdf]

Additional file 1 – Documentary Evidence: List of reports and documents cited by CIs and EUs (excluded peer review publications)

1. Abbott RA, Macdonald D, Mackinon L, Stubbs C, Lee AJ, Harper C *et al*.. Healthy kids Queensland survey 2006 - summary report. 2007. Brisbane, Queensland Health.
2. Booth ML, Okely AD, Denney-Wilson E, Hardy L, Yang B, Dobbins T. NSW Schools Physical Activity and Nutrition Survey (SPANS) 2004: Full Report. 2006. Sydney, NSW Department of Health.
3. Booth ML, Okely AD, Denney-Wilson E, Hardy LL, Yang B, Dobbins T. NSW Schools Physical Activity and Nutrition Survey (SPANS) 2004: Summary Report. 2006. Sydney, NSW Department of Health.
4. Booth ML, Okely AD, Denney-Wilson E, Hardy LL, Yang B, Dobbins T. NSW Schools Physical Activity and Nutrition Survey (SPANS) 2004: Short Report. 2006. Sydney, NSW Department of Health.
5. Booth M, Macaskill P, McLellan L, Phongsavan P, Okely T, Patterson J *et al*.. NSW Schools Fitness and Physical Activity Survey 1997. 1997. Ryde, NSW Department of Education and Training.
6. Hardy LL, King L, Espinel P, Cosgrove C, Bauman A. NSW Schools Physical Activity and Nutrition Survey 2010: Executive Summary. 2011.
7. Hardy LL, King L, Espinel P, Cosgrove C, Bauman A. NSW Schools Physical Activity and Nutrition Survey (SPANS) 2010: Full Report. 2011. Sydney, NSW Ministry of Health.
8. Hardy LL, King L, Espinel P, Cosgrove C, Bauman A. NSW Schools Physical Activity and Nutrition Survey (SPANS) 2010:Short Report. 2011. Sydney, NSW Ministry of Health.
9. Hunter New England Area Health Service, NSW Department of Health, Physical Activity Nutrition Obesity Research Group. Good for Kids, Good for Life. 2012.
10. Martin K, Rosenberg M, Millar M, French S, McCormack G, Bull F *et al*.. Move and Munch Final Report. Trends in physical activity, nutrition and body size in Western Australian children and adolescents: the Child and Adolescent Physical Activity and Nutrition Survey (CAPANS). 2008. Perth, Western Australian Government.
11. NSW Department of Health, NSW Department of Education and Communities., Heart Foundation.. Healthy Kids: Eat well, get active. 2012.
12. NSW Government.. NSW Government Action Plan 2003-2007 - Prevention of Obesity in Children and Young People. 2003. Sydney, NSW Department of Health.
13. Morley B, Scully M, Niven P, Wakfield M. National Secondary Students' Diet and Physical Activity Survey 2009-10: Main Report. 2012. Melbourne, Cancer Council Victoria.
14. NSW Department of Education and Training. Get skilled: Get active. A K-6 resource to support the teaching of fundamental movement skills. 2000. Ryde, NSW Department of Education and Training.
15. Wright J, Konza D, Hearne D, Okely T: The Gold Medal Fitness Program: a model for teacher change'. *Physical Education & Sport Pedagogy* 2008, **13:** 49-64.

Documentary Evidence: List of SPANS peer review publications

2010 survey

1. Hardy LL, King L, Espinel P, Okely AD, Bauman A. Methods of the NSW Schools Physical Activity and Nutrition Survey 2010 (SPANS 2010). J Sci Med Sport 2011 March 30
2. Meron D, Rissel C, Reinten-Reynolds T, Hardy LL. Changes in active travel of school children from 2004 to 2010 in New South Wales, Australia. *Prev Med 2011*.
3. Rissel CE, Reinten-Reynolds TA, Wen LM, Hardy LL. Soft drink consumption and obesity in NSW school students. *Med J Aust* 2012;196:171-172.
4. Hardy LL, Cosgrove C, King L, Venugopal K, Baur LA, Gill T. Shifting curves? Trends in thinness and obesity among Australian youth, 1985 to 2010. Pediatr Obes 2012;7:92-100.
5. Hardy LL, Reinten-Reynolds T, Espinel P, Zask A, Okely AD: Prevalence and Correlates of Low Fundamental Movement Skill Competency in Children. Pediatrics 2012.

2004 survey

1. Booth ML, Okely AD, Denney-Wilson E. Validation and application of a novel method of measuring non-response bias in school-based surveys of paediatric overweight and obesity. *Int J Pediatr Obes* 2011; 6(2-2):e87-e93.
2. Booth ML, Denney-Wilson E, Okely AD, Hardy LL. Methods of the NSW Schools Physical Activity and Nutrition Survey (SPANS). *J Sci Med Sport* 2005; 8(3):284-293.
3. Booth ML, Dobbins T, Okely AD, Denney-Wilson E, Hardy LL. Trends in the prevalence of overweight and obesity among young Australians, 1985, 1997, and 2004. *Obesity (Silver Spring)* 2007; 15(5):1089-1095.
4. Booth ML, Okely AD, Denney-Wilson E, Hardy LL, Dobbins T, Wen LM et al. Characteristics of travel to and from school among adolescents in NSW, Australia. *J Paediatr Child Health* 2007; 43(11):755-761.
5. Booth ML, George J, Denney-Wilson E, Okely AD, Hardy LL, Aitken R et al. The population prevalence of adverse concentrations and associations with adiposity of liver tests among Australian adolescents. *J Paediatr Child Health* 2008; 44(12):686-691.
6. Booth ML, Dobbins T, Aitken R, Denney-Wilson E, Hardy LL, Okely AD et al. Costs of managing conditions associated with obesity among Australian teenagers. *J Paediatr Child Health* 2009; 45(7-8):448-456.
7. Denney-Wilson E, Hardy LL, Dobbins T, Okely AD, Baur LA. Body mass index, waist circumference, and chronic disease risk factors in Australian adolescents. *Arch Pediatr Adolesc Med* 2008; 162(6):566-573.
8. Denney-Wilson E, Kenny DT, Hardy LL, Nelson P. Associations between overweight and obesity and risk factors for cardiovascular disease and fatty liver in young offenders serving community orders. *Vulnerable Children and Youth Studies* 2007; 2(2):165-172.

9. Denney-Wilson E, Crawford D, Dobbins T, Hardy L, Okely AD. Influences on consumption of soft drinks and fast foods in adolescents. *Asia Pac J Clin Nutr* 2009; 18(3):447-452.

10. Denney-Wilson E, Cowell CT, Okely AD, Hardy LL, Aitken R, Dobbins T. Associations between insulin and glucose concentrations and anthropometric measures of fat mass in Australian adolescents. *BMC Pediatr* 2010; 10:58.

11. George J, Denney-Wilson E, Okely AD, Hardy LL, Aitken R. The population distributions, upper normal limits and correlations between liver tests among Australian adolescents. *J Paediatr Child Health* 2008; 44(10):579-585.

12. Hardy LL, Dobbins TA, Denney-Wilson E, Okely AD, Booth ML. Descriptive epidemiology of small screen recreation among Australian adolescents. *J Paediatr Child Health* 2006; 42(11):709-714.

13. Hardy LL, Dobbins T, Booth ML, Denney-Wilson E, Okely AD. Sedentary behaviours among Australian adolescents. *Aust N Z J Public Health* 2006; 30(6):534-540.

14. Hardy LL, Okely AD, Dobbins TA, Booth ML. Physical activity among adolescents in New South Wales (Australia): 1997 and 2004. *Med Sci Sports Exerc* 2008; 40(5):835-841.

15. Hardy LL, Dobbins TA, Denney-Wilson E, Okely AD, Booth ML. Sedentariness, small-screen recreation, and fitness in youth. *Am J Prev Med* 2009; 36(2):120-125.

16. Hardy LL, Denney-Wilson E, Thrift AP, Okely AD, Baur LA. Screen Time and Metabolic Risk Factors Among Adolescents. *Arch Pediatr Adolesc Med* 2010; 164(7):643-649.

17. Okely AD, Booth ML, Hardy L, Dobbins T, Denney-Wilson E. Changes in physical activity participation from 1985 to 2004 in a statewide survey of Australian adolescents. *Arch Pediatr Adolesc Med* 2008; 162(2):176-180.

18. Okely AD, Hardy LL, Booth ML, Dobbins TA, Denney-Wilson EA, Yang B. Changes in cardiorespiratory fitness among children and adolescents in Australia: 1997 and 2004. *Journal of Sports Sciences* 2010; 28(8):851-857.

1997 survey

1. Booth ML, Macaskill P, Phongsavan P, McLellan L, Okely T. Methods of the NSW Schools Fitness and Physical Activity Survey, 1997. J Sci Med Sport 1998; 1(2):111-124.
2. Booth ML, Chey T, Wake M, Norton K, Hesketh K, Dollman J et al. Change in the prevalence of overweight and obesity among young Australians, 1969-1997. Am J Clin Nutr 2003; 77(1):29-36.
3. Booth ML, Okely AD, Chey T, Bauman AE, Macaskill P. Epidemiology of physical activity participation among New South Wales school students. Aust N Z J Public Health 2002; 26(4):371-374.

4. Booth ML, Wake M, Armstrong T, Chey T, Hesketh K, Mathur S. The epidemiology of overweight and obesity among Australian children and adolescents, 1995-97. Aust N Z J Public Health 2001; 25(2):162-169.
5. Booth ML, Okely T, McLellan L, Phongsavan P, Macaskill P, Patterson J et al. Mastery of fundamental motor skills among New South Wales school students: prevalence and sociodemographic distribution. J Sci Med Sport 1999; 2(2):93-105.
6. Booth ML, Macaskill P, Lazarus R, Baur LA. Sociodemographic distribution of measures of body fatness among children and adolescents in New South Wales, Australia. Int J Obes Relat Metab Disord 1999; 23(5):456-462.
7. Okely AD, Booth ML, Chey T. Relationships between body composition and fundamental movement skills among children and adolescents. Res Q Exerc Sport 2004; 75(3):238-247.
8. Okely AD, Booth ML, Patterson JW. Relationship of physical activity to fundamental movement skills among adolescents. Med Sci Sports Exerc 2001; 33(11):1899-1904.
